# Recent Developments in the [1,2]-Phospha-Brook Rearrangement Reaction

**DOI:** 10.3390/ijms26073065

**Published:** 2025-03-27

**Authors:** Ning Li, Qian Wu, Yu Huang, Enxue Shi, Junchen Li

**Affiliations:** State Key Laboratory of NBC Protection for Civilian, Beijing 102205, China; ln1622126@163.com (N.L.); wuqian11210922@163.com (Q.W.); 13597628349@163.com (Y.H.); exshi@sina.com (E.S.)

**Keywords:** [1,2]-phospha-Brook rearrangement, carbonyl compounds, phosphodiesters

## Abstract

The [1,2]-phospha-Brook rearrangement serves as a powerful synthetic strategy that enables efficient carbonyl umpolung through phosphoryl group migration, providing direct access to α-hydroxyphosphoryl compounds—a privileged class of synthons with broad applications in organophosphorus chemistry, medicinal chemistry, and materials science. This review provides a comprehensive overview of recent progress in synthetic methodologies, possible mechanisms, and asymmetric transformations, highlighting key breakthroughs and future directions in this rapidly evolving field.

## 1. Introduction 

**Figure 1:**
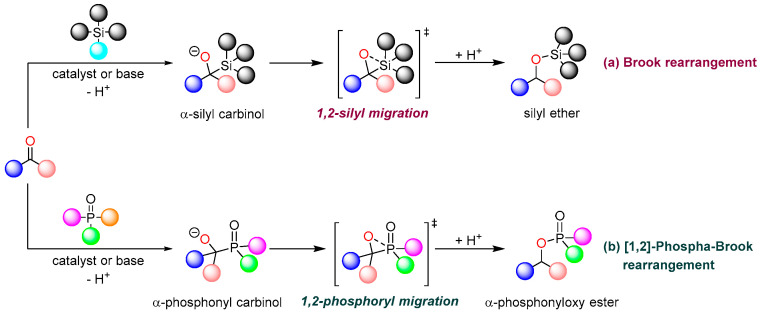
Brook rearrangement and [1,2]-phospha-Brook rearrangement. (**a**) Brook rearrangement. (**b**) [1,2]-phospha-Brook rearrangement.

The Brook rearrangement, also known as the intramolecular 1,2-silicon-based migration reaction, was first reported by Brook in 1958 [1]. This reaction typically involves an intramolecular 1,2-anionic migration of a silyl group in an α-silyl carbinol intermediate, which is generated from a silicon reagent and a carbonyl substrate. The process proceeds through the formation of a cyclic silicon species, facilitated by catalysts or bases, ultimately yielding the corresponding silyl ether as the product (Figure 1a) [2]. The [1,2]-phospha-Brook rearrangement represents a variant of the Brook rearrangement, characterized by the substitution of the original silicon atom with a phosphorus atom as the migratory center. This transformation specifically involves the rearrangement reaction of α-hydroxyphosphonates, where the phosphonyl group migrates from a carbon atom to an oxygen atom, generating α-phosphonyloxy ester as the product (Figure 1b). The [1,2]-phospha-Brook rearrangement serves as a valuable synthetic tool for the efficient preparation of biologically active α-phosphoryloxy esters, which find extensive applications in medicine, materials science, pesticide development, and the total synthesis of natural products. Notably, the α-phosphoryloxy structure readily generates carbon anions that can participate in coupling reactions to form new C-C bonds, thereby providing a versatile platform for constructing complex molecular architectures, particularly compounds with multiple chiral centers.

Terada and coworkers [3,4] have reviewed the [1,2]-phospha-Brook rearrangement for generating anionic nucleophiles in addition reactions, while Singh [5] has focused on stereoselective or enantioselective transformations of various structural units. However, a comprehensive classification based on carbonyl substrate types remains unexplored. This review summarizes the progress of [1,2]-phospha-Brook rearrangement over the past two decades. The content is organized into several sections based on the types of carbonyl substrates involved in the reaction, including aldehydes, ketones, ketoesters, ketoamides, vinyl ketones, alkynyl ketones, and α-hydroxyphosphates.

## 2. Aldehyde Rearrangement

Although the use of simple aldehyde substrates in Pudovik reactions has been extensively documented, their application in [1,2]-phospha-Brook rearrangement reactions remains relatively unexplored. This limited exploration can be attributed to the significantly higher activation energy barrier associated with the [1,2]-phospha-Brook rearrangement compared to the Pudovik reaction, which necessitates more rigorous reaction conditions. These demanding thermodynamic requirements have substantially hindered progress in this research area. It is noteworthy that while aldehyde substrates have occasionally been employed in certain [1,2]-phospha-Brook reaction studies, they have primarily served supplementary roles within experimental frameworks rather than being the primary focus of investigation.

### 2.1. Aldehyde Rearrangement with Phosphodiesters

**Figure 2:**
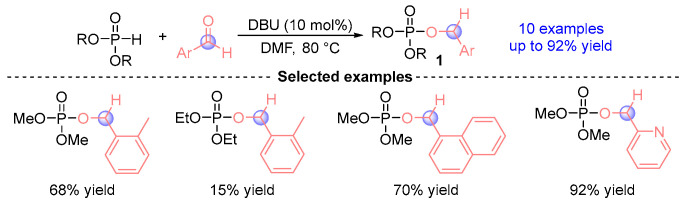
DBU-catalyzed [1,2]-phospha-Brook rearrangement reported by Kaïm.

In 2005, Kaïm et al. [6] reported a pioneering discovery wherein phosphodiesters reacted with aromatic aldehydes in DMF (*N*,*N*-dimethylformamide) mediated by the organic base DBU (1,8-diazabicyclo [5.4.0]undec-7-ene), affording α-phosphoryloxy ester derivatives **1** (Figure 2). This breakthrough emerged against the backdrop of previous limitations in [1,2]-phospha-Brook rearrangements, where such transformations with carbonyl compounds were predominantly confined to specialized substrates featuring anion stabilizing α-substituents including α-dicarbonyl compounds, perfluoroalkyl aldehydes/ketones, benzophenones, cyclopentadienones, and typically required low-temperature conditions. Notably, this work represents the inaugural documented [1,2]-phospha-Brook rearrangement employing simple aldehydes as coupling partners, achieving remarkable synthetic efficiency (up to 92% yield) across a diverse set of 10 derivatives. These findings established crucial mechanistic and synthetic precedents for subsequent advancements in aldehyde-involved [1,2]-phospha-Brook rearrangement chemistry.

**Figure 3:**
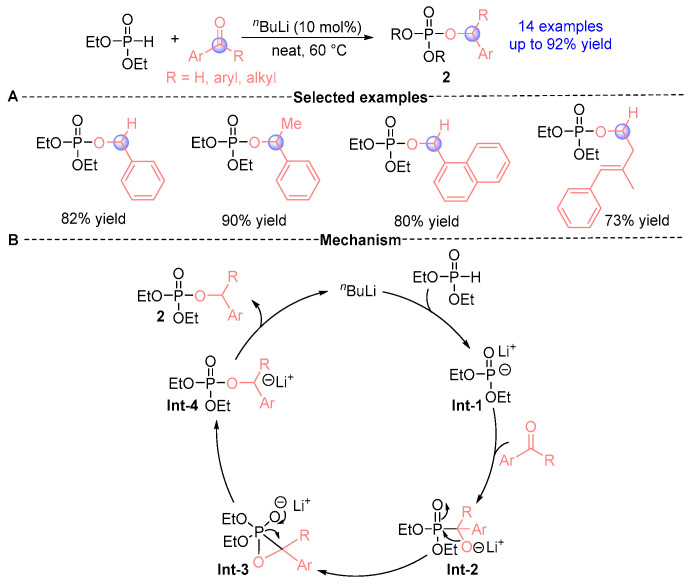
*^n^*BuLi-catalyzed [1,2]-phospha-Brook rearrangement reported by Manab. (**A**) Selected examples for **2a**. (**B**) Plausible mechanism.

In 2015, Manab [7] demonstrated that diethyl phosphite under *^n^*BuLi (*n*-butyllithium) catalysis could undergo efficient rearrangement with aldehydes under solvent-free conditions, establishing a robust synthetic protocol. Building upon this foundation, Ghosal’s research group [8] subsequently optimized the methodology in 2019, achieving remarkable synthetic efficiency (up to 92% yield) across structurally distinct α-phosphoryloxy ester derivatives **2** (Figure 3A). This innovative approach enabled the streamlined synthesis of two valuable compound classes: (i) allyl phosphate esters exhibiting notable bioactivity and broad synthetic utility, and (ii) electron-rich ketones bearing strategically positioned electron-donating substituents. Mechanistic investigations were systematically conducted to elucidate the reaction pathway (Figure 3B). The proposed catalytic cycle initiates with Li⁺-mediated generation of intermediate **Int-1** from diethyl phosphite, followed by a Pudovik addition with carbonyl compounds to form **Int-2**. This α-hydroxyphosphonate intermediate undergoes sequential transformation through a metastable ternary transition state (**Int-3**) prior to final reorganization into product **2** via **Int-4**. Notably, comprehensive reaction monitoring revealed substrate-dependent behavior: α-hydroxyphosphonates (**Int-2**) were isolable in aldehyde-based reactions but proved elusive with ketone substrates. To decipher this substrate dichotomy, DFT (density functional theory) calculations unveiled critical energy landscape features. Crucially, the formation of **Int-3** from benzaldehyde-derived **Int-2** exhibited a substantial 10 kcal/mol energy barrier elevation compared to benzophenone analogs, accounting for the slower **Int-2**→**Int-4** conversion kinetics in aldehyde systems. This kinetic disparity rationalizes the experimentally observed isolability of α-hydroxyphosphonates exclusively in aldehyde reactions, while explaining the inherent predisposition of ketone substrates toward facile [1,2]-phospha-Brook rearrangement to directly yield α-phosphoryloxy esters.

**Figure 4:**
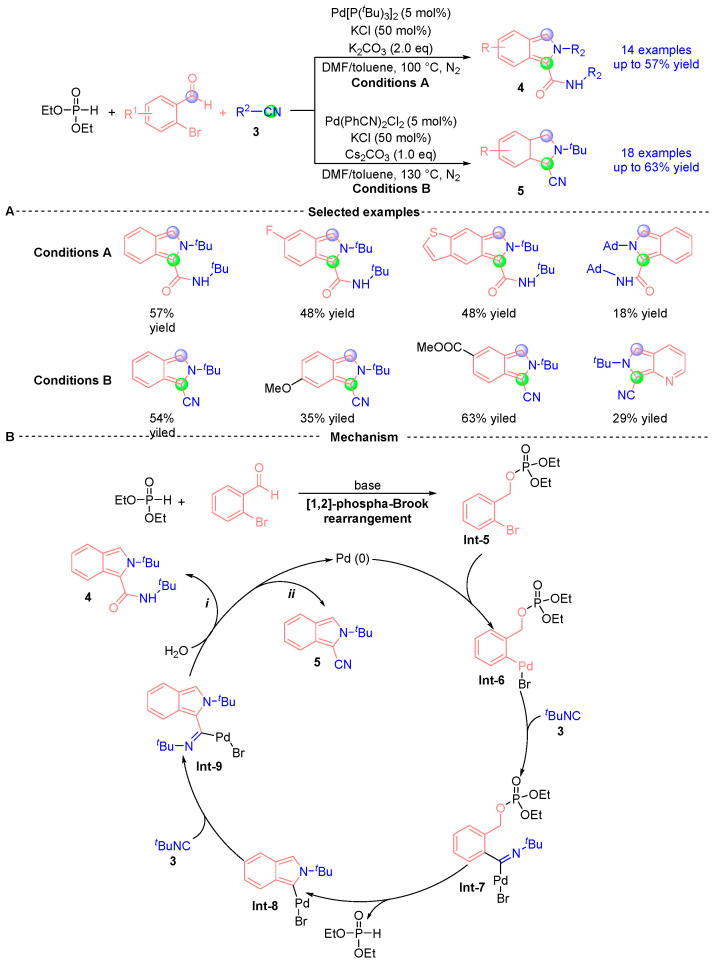
Pd-catalyzed [1,2]-phospha-Brook rearrangement reported by Wu. (**A**) Selected examples for **4** and **5**. (**B**) Plausible mechanism.

Recently, Wu’s group [9] reported a palladium-catalyzed cyclization reaction leveraging [1,2]-phospha-Brook rearrangement to achieve base-modulated chemodivergent synthesis of 2*H*-isoindole-1-carboxamide **4** and 2*H*-isoindole-1-carbonitrile **5** derivatives (Figure 4A). This methodology exhibits three hallmark characteristics: (i) dual isocyanide incorporation, (ii) concurrent C-C/C-N bond formation, and (iii) ambient-temperature operability. Mechanistic investigations (Figure 4B) demonstrate the catalytic cycle commences with base-mediated [1,2]-phospha-Brook rearrangement between *o*-bromobenzaldehyde and diethyl phosphite, yielding phosphoryl intermediate **Int-5**. Subsequent oxidative addition of the C-Br bond to Pd(0) generates organopalladium species **Int-6**, which undergoes first isocyanide **3** migratory insertion to afford iminopalladium(II) bromide **Int-7**. This intermediate progresses through intramolecular cyclization/isomerization to form metallocycle **Int-8**, followed by secondary isocyanide insertion producing Pd(II) intermediate **Int-9**. Divergent termination pathways emerge through either: (i) aqueous nucleophilic capture of **Int-9** yielding carboxamide **4,** or (ii) β-carbon elimination en route to carbonitrile **5**, while accomplishing catalytic regeneration of Pd(0) for sustained turnover.

### 2.2. Aldehyde Rearrangement with Phosphodiester Precursors

**Figure 5:**
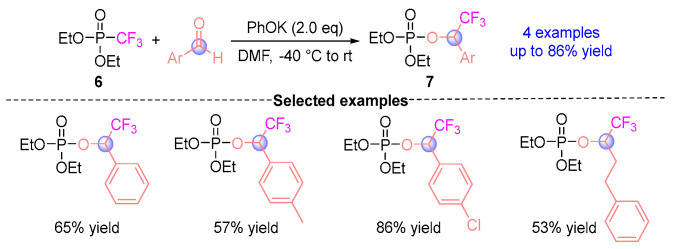
PhOK-mediated [1,2]-phospha-Brook rearrangement reported by Petr.

In 2010, Petr et al. [10] developed a pioneering trifluoromethylation protocol through [1,2]-phospha-Brook rearrangement (Figure 5), producing α-CF_3_-phosphoryloxy esters **7** via coupling diethyl trifluoromethylphosphate **6** with aromatic aldehydes under alkoxide base mediation (*^t^*BuOK (potassium *tert*-butoxide)/PhOK (potassium phenoxide)). This work established a catalytic platform for electrophilic trifluoromethylation, demonstrating remarkable functional group compatibility while unlocking innovative trifluoromethylation strategies for diverse electrophilic substrates.

### 2.3. Aldehyde Rearrangement with Secondary Phosphine Oxides

**Figure 6:**
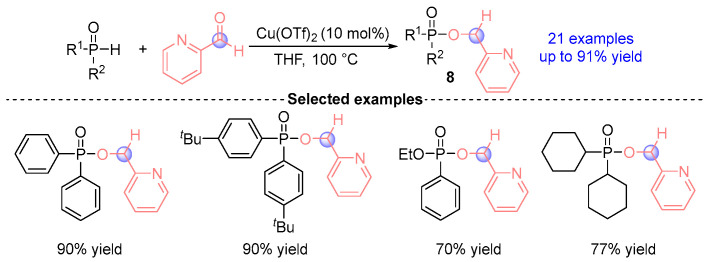
Cu(OTf)_2_-catalyzed [1,2]-phospha-Brook rearrangement reported by Yang.

Yang and coworkers [11] established in 2022 a Lewis acid-catalyzed paradigm for [1,2]-phospha-Brook rearrangements, employing Lewis acid Cu(OTf)_2_ to mediate reactions between secondary phosphine oxides (SPOs) and aldehydes (Figure 6). This catalytic system generated 38 pyridine-derived α-phosphoryloxy architectures alongside their dimeric bisphosphoryloxy derivatives **8** with unprecedented 94% efficiency. The methodology demonstrated excellent functional tolerance across diverse phosphine oxide donors and pyridine-based electrophiles, thereby significantly expanding the synthetic versatility of [1,2]-phospha-Brook rearrangement in organophosphorus chemistry.

### 2.4. Aldehyde Rearrangement with SPO-Modified Precursors

**Figure 7:**
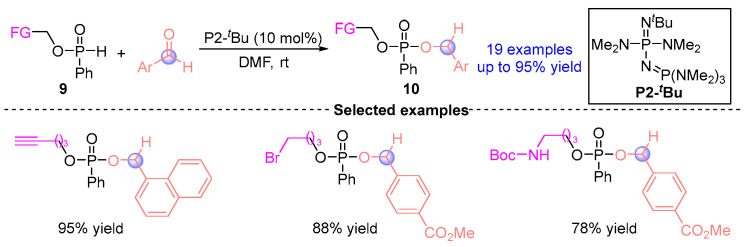
P2-*^t^*Bu-catalyzed [1,2]-phospha-Brook rearrangement reported by Terda.

In 2022, Terda [12] disclosed the [1,2]-phospha-Brook reaction of SPO-modified precursor **9** (alkynyl, bromoalkyl, *N*-Boc amino, and boron-based phosphonates) catalyzed by Brønsted base P2-*^t^*Bu with aldehydes, successfully obtaining 19 corresponding functionalized phosphonates **10** (Figure 7). The methodology established a novel molecular editing strategy for post-synthetic functionalization of carbonyl-containing scaffolds, particularly enabling precise functional group installation at ketone or formyl anchoring sites.

## 3. Ketone Rearrangement

Comparative analysis reveals distinct activation profiles between carbonyl substrates in [1,2]-phospha-Brook rearrangements. Ketone derivatives exhibit reduced activation barriers relative to aldehyde counterparts, enabling transformations to proceed with enhanced kinetic facility. Notably, electron-deficient ketones bearing strong electron-withdrawing substituents demonstrate superior reaction efficiency through transition state stabilization. Within this mechanistic framework, acyl phosphonates are categorized as privileged substrates due to their reactive phosphoryl centers. These compounds fundamentally undergo [1,2]-phospha-Brook rearrangement to generate stabilized carbanion intermediates that engage in downstream derivatization. Significantly, this substrate class displays a unique propensity for intramolecular rearrangement pathways distinct from classical intermolecular variants.

### 3.1. Ketone Rearrangement with Phosphodiester Precursors

**Figure 8:**
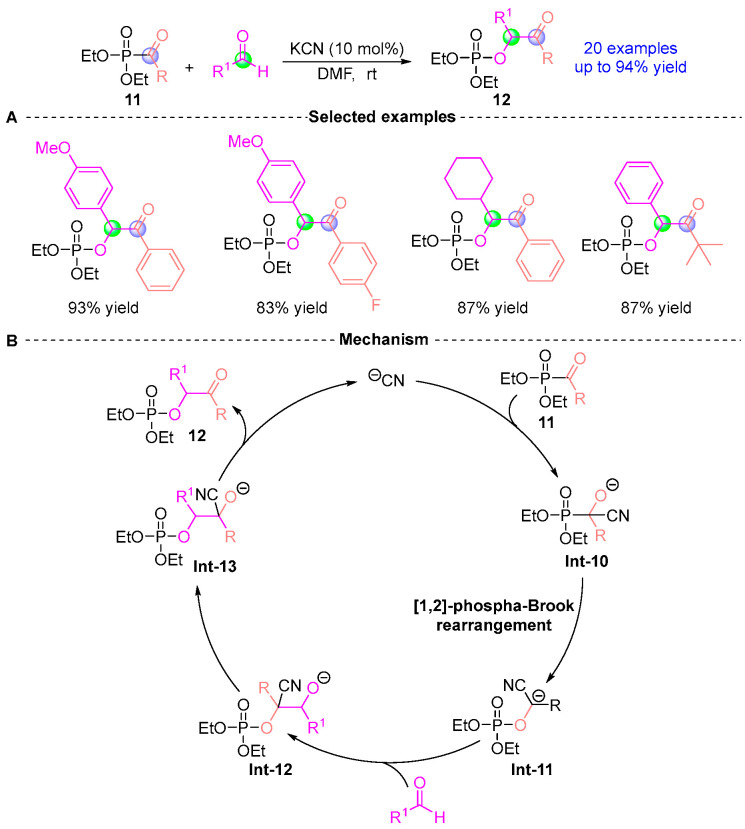
KCN-catalyzed [1,2]-phospha-Brook rearrangement reported by Eymur. (**A**) Selected examples for **12**. (**B**) Plausible mechanism.

In 2005, Eymur [13] disclosed that under the catalytic effect of KCN, acylphosphonic acid ester **11** could react with aldehydes to obtain cross benzobenzoin product **12** with outstanding yield and regioselectivity (Figure 8A). The proposed catalytic cycle (Figure 8B) initiates with cyanide-mediated nucleophilic activation of compound **11**, generating cyanophosphate alkoxide intermediate **Int-10**. Subsequent [1,2]-phospha-Brook rearrangement produces stabilized acyl anion equivalent **Int**-**11**, which engages in carbonyl addition to form tetrahedral adduct **Int-12**. Phosphorylation of this intermediate yields **Int-13,** ultimately undergoing protonolysis to release product **12**. Relative to conventional acylsilane-mediated protocols, this mechanistically distinct strategy demonstrates enhanced atom economy and accelerated reaction kinetics, establishing an operationally simple platform for catalytic carbanion generation.

**Figure 9:**
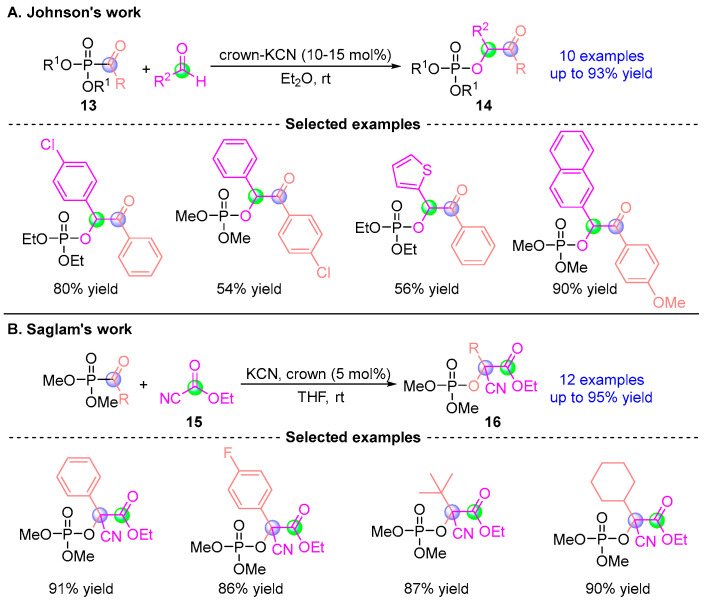
KCN/crown-catalyzed [1,2]-phospha-Brook rearrangement reported by Johnson and Saglam. (**A**) Johnson’s crown-KCN catalytic method. (**B**) Saglam’s crown/KCN catalytic method.

Building upon this foundation, Johnson [14] developed an enhanced catalytic system employing crown ether-modified KCN to mediate reactions between acyl phosphonates and aldehydes, producing α-phosphoryloxy esters **14** in up to 93% yields (Figure 9A). Remarkably, these products underwent chemoselective P–O bond cleavage in aqueous amine solutions to afford chiral benzoic acid derivatives, demonstrating exceptional configurational stability. Subsequently, Saglam’s team [15] engineered a cyanide-catalyzed protocol in 2007 for a one-pot synthesis of cyano-functionalized α-phosphoryloxy esters **16** (Figure 9B). These compounds served as versatile synthons for constructing diverse bioactive scaffolds, including privileged structures in medicinal chemistry.

### 3.2. Ketone Rearrangement with Phosphodiesters

**Figure 10:**
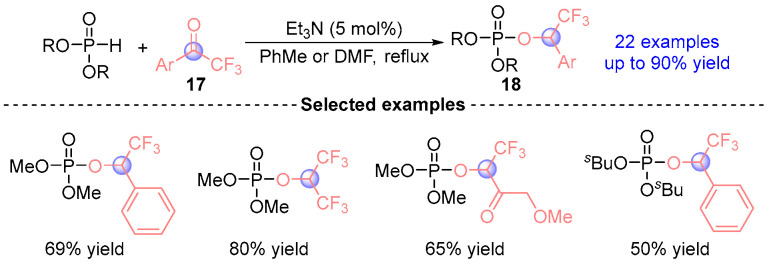
Et_3_N-catalyzed [1,2]-phospha-Brook rearrangement reported by Serebryakova and Makhaeva.

Consecutive studies by Serebryakova [16] in 2009 and Makhaeva [17] in 2010 established catalytic protocols for synthesizing α-phosphoryloxy trifluoromethyl esters **18** via [1,2]-phospha-Brook rearrangements (Figure 10). Employing Et_3_N mediated reactions between trifluoromethyl ketones **17** and phosphodiesters under thermal conditions mechanistic investigations revealed initial Pudovik addition yielding α-hydroxyphosphonates, followed by thermal activation driving rearrangement to thermodynamically favored α-phosphoryloxy products. The pharmacological evaluation demonstrated compound **18**’s pronounced selectivity as AChE (acetylcholinesterase)and BChE (butyrylcholinesterase) inhibitor with phenyl-substituted derivatives exhibiting reversible CaE (carboxylesterases) inhibition. Notably, electronic activation parameters confirmed electron-withdrawing α-substituents as critical structural determinants.

**Figure 11:**
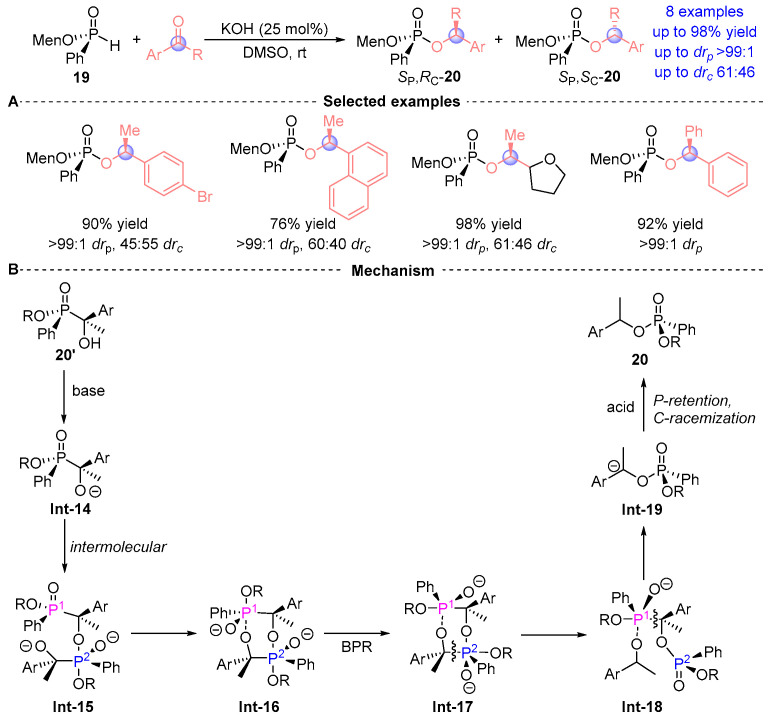
KOH-catalyzed [1,2]-phospha-Brook rearrangement reported by Zhao. (**A**) Selected examples for **20**. (**B**) Plausible mechanism.

Zhao et al. [18] pioneered the first chiral auxiliary-mediated [1,2]-phospha-Brook rearrangement system, employing menthol-derived *H*-phosphonate (Menthyl-PHO) **19** with ketone substrates (Figure 11A). While demonstrating broad compatibility with aryl aldehydes, the methodology exhibits notable substrate scope constraints for ketones, yielding merely 10 derivatives **20**. Mechanistic analysis revealed complete configuration retention at the phosphorus center (>99:1 *dr_p_*) but limited stereochemical induction at the α-carbon (highest reach to 61:46 *dr_c_*), suggesting partial erosion of chiral information transfer during the rearrangement process. This seminal work delineates a groundbreaking intermolecular mechanism for [1,2]-Phospha-Brook rearrangement, representing a paradigm shift from classical intramolecular pathways (Figure 11B). The catalytic cycle commences with base-induced deprotonation of **20’** to generate oxyanion **Int-14**, which orchestrates nucleophilic displacement at phosphorus in a second substrate molecule, forming dimeric intermediate **Int-15**. Subsequent cyclization via O→P transesterification produces chair-configured **Int-16**, where stereochemical inversion occurs through Berry pseudorotation (BPR) to axial **Int-17**. Concerted bond reorganization cleaves Cα-P^2^ yield **Int-18** followed by Cα-P^1^ dissociation **Int-19**, ultimately delivering product **20** via protonation. Crucially, phosphorus configuration remains preserved despite complete stereochemical scrambling at Cα, demonstrating unprecedented mechanistic divergence from conventional systems.

## 4. Ketoester Rearrangement

Ketoesters constitute privileged synthons in contemporary organic synthesis, finding extensive application as pivotal intermediates for pharmaceutical development and complex molecule construction. Owing to their superior reactivity compared to simple ketones, these compounds exhibit exceptional utility in [1,2]-phospha-Brook rearrangements.

### 4.1. Ketoester Rearrangement with Phosphodiesters

**Figure 12:**
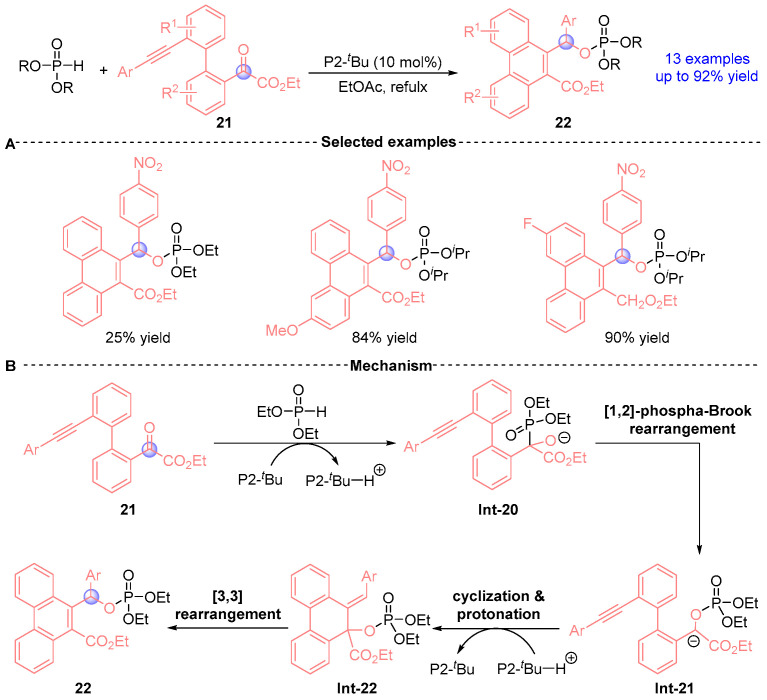
P2-*^t^*Bu-catalyzed [1,2]-phospha-Brook rearrangement reported by Terada. (**A**) Selected examples for **22**. (**B**) Plausible mechanism.

Terada et al. [19] demonstrated in 2015 a Brønsted base-catalyzed annulation strategy utilizing phosphazene base P2-*^t^*Bu to synthesize functionalized phenanthrenes 22 through sequential [1,2]-phospha-Brook/[3,3]-sigmatropic rearrangements (Figure 12A). This transformation proceeds through: (i) base-mediated Pudovik addition between phosphodiester and ketoester yield **Int-20**, (ii) [1,2]-phospha-Brook rearrangement generating stabilized carbanion intermediate **Int-21**, (iii) intramolecular cyclization forming **Int-22** with chair-like transition state, and (iv) [3,3]-sigmatropic reorganization delivering phenanthrene derivatives **22** (Figure 12B).

**Figure 13:**
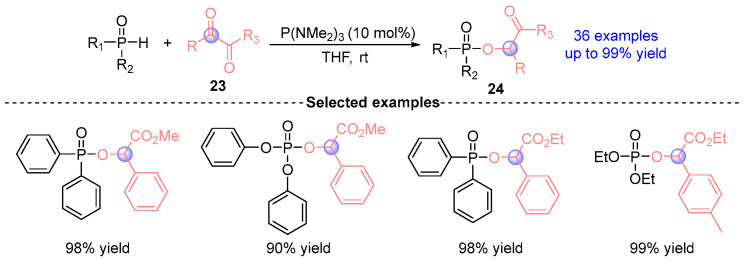
P(NMe_2_)_3_-catalyzed [1,2]-phospha-Brook rearrangement reported by Zi.

In 2023, Zi’s group [20] demonstrates a catalytic *O*-phosphination strategy for α-dicarbonyl derivatives **24** through sequential Kukhtin–Ramirez adduct formation and P(NMe₂)₃-mediated ambiphilic activation (Figure 13). This versatile platform accommodates diverse P(O)–H nucleophiles (diarylphosphine oxides, arylphosphinates, phosphinates) with high yields across >30 substrates, achieving gram-scale synthesis. The operational simplicity, paired with water tolerance and air stability, establishes this methodology as a robust platform for late-stage phosphorylation of bioactive carbonyls.

**Figure 14:**
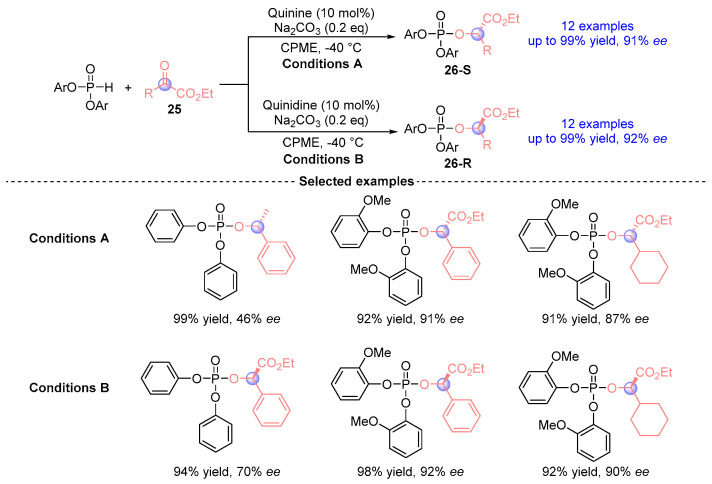
Quinine or quinidine-catalyzed [1,2]-phospha-Brook rearrangement reported by Nakamur.

Nakamura et al. [21] achieved a milestone by developing the first stereodivergent synthesis of α-phosphoryloxy esters **26** through quinine or quinidine catalyzed [1,2]-phospha-Brook rearrangements in 2011. Employing a chiral Lewis base catalysis system (quinine/Na_2_CO_3_) with phosphodiesters and α-ketoesters **25**, this protocol delivered **26** derivatives up to 99% yield with up to 92% *ee* (Figure 14). Stereochemical control arises from the precise manipulation of alkaloid substituents, enabling predictable configuration inversion at Cα. Mechanistic studies revealed that enantioselectivity originates from enantiotopic face discrimination during enol protonation.

### 4.2. Ketoester Rearrangement with Three Components

**Figure 15:**
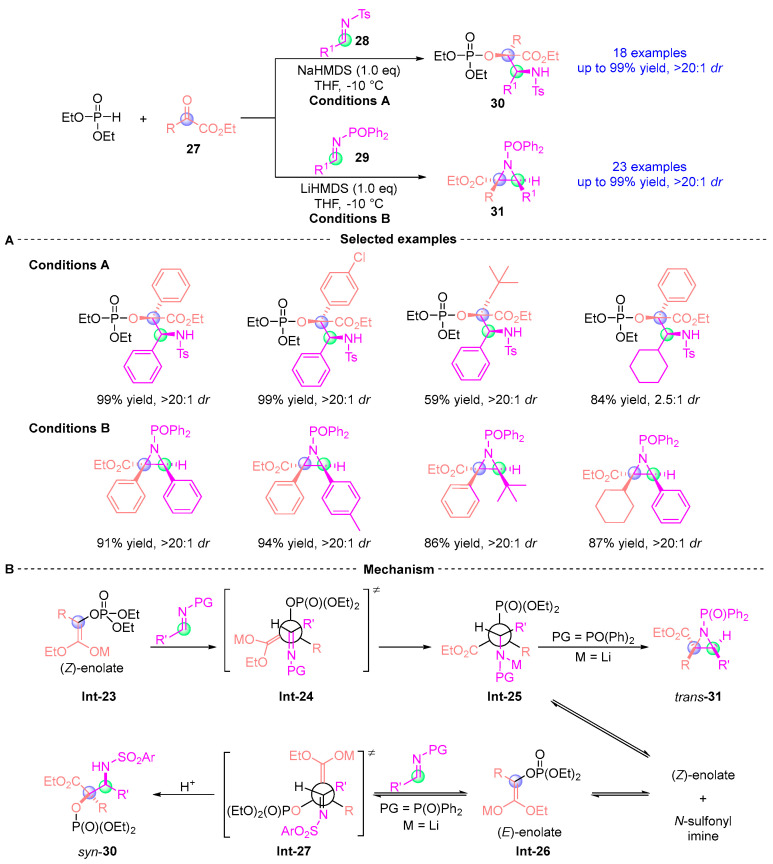
Li(Na)HMDS-mediated [1,2]-phospha-Brook rearrangement reported by Xu. (**A**) Selected examples for **30** and **31**. (**B**) Plausible mechanism.

In 2016, Xu [22] disclosed a catalyst-free stereodivergent synthesis through LiHMDS (lithium hexamethyldisilazide)) or NaHMDS (sodium hexamethyldisilazide) mediated three-component cascades of α-ketoesters **27**, phosphites, and imines (**28**–**29**). This transformation orchestrates: (i) [1,2]-phospha-Brook rearrangement, (ii) stereoselective Mannich addition (up to 20:1 *dr*), and (iii) *aza*-Darzens cyclization, delivering *syn*-α-hydroxy-β-amino acids **30** and *trans*-aziridines **31** through switchable pathways. Mechanistic analysis revealed *N*-protecting group-dependent stereocontrol: sulfonyl imines direct (*Z*)-enol **Int-23** to open **Int-24**, which then evolves into **Int-25**, followed by 3-*exo* cyclization to yield *trans*-aziridine **31**. In contrast, bulky *N*-aryl groups promote the formation of (*E*)-enol **Int-26** via retro-Mannich/tautomerization cascades, ultimately leading to product **30** (Figure 15).

**Figure 16:**
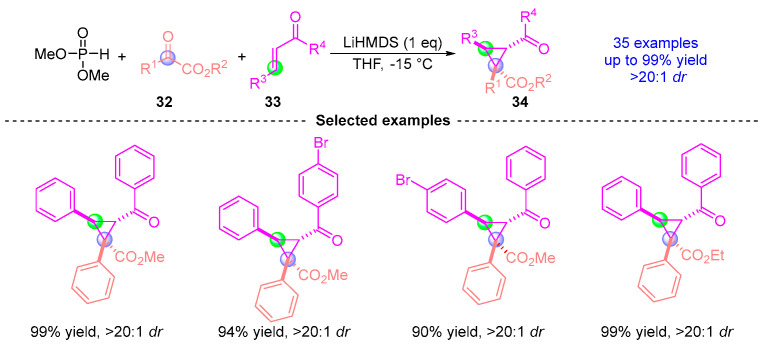
LiHMDS-promoted [1,2]-phospha-Brook rearrangement reported by Xu.

Building upon their earlier work, Xu [23] reported in 2017 an efficient and highly selective method for the asymmetric synthesis of chiral cyclopropane compounds via [1,2]-phospha-Brook rearrangement (Figure 16). This three-component coupling reaction employs dimethyl phosphite, ketoester **32**, and α,β-unsaturated ketones **33** as starting materials. Under LiHMDS catalysis, the reaction proceeds through a stereoselective pathway to yield highly functionalized cyclopropane compound **34** with excellent diastereoselectivity (*dr* > 20:1) and high yield (up to 90%).

**Figure 17:**
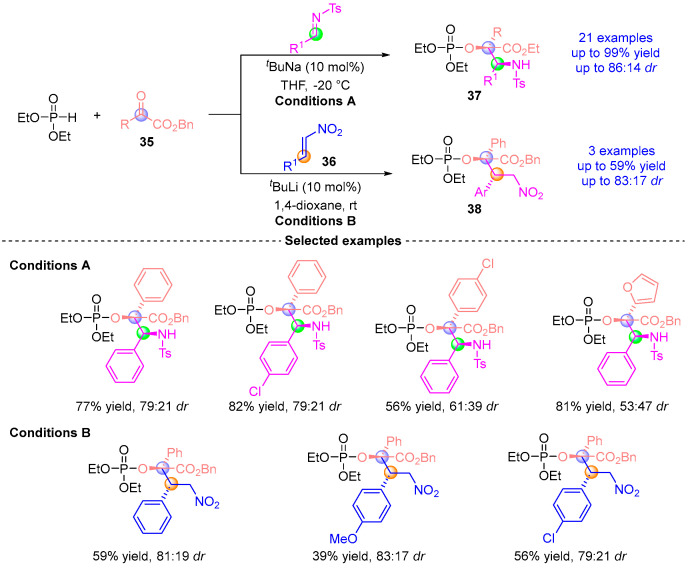
*^t^*BuNa(Li)-catalyzed [1,2]-phospha-Brook rearrangement reported by Terada.

In 2016, Terada’s group [24] developed a three-component asymmetric coupling reaction involving α-ketoester **35**, diethyl phosphite, and either imine or nitroalkene **36** (Figure 17). This catalytic system, based on the same principle, proceeds through a cascade of Pudovik addition and [1,2]-phospha-Brook rearrangement under the catalysis of NaO*^t^*Bu/LiHMDS, generating an enol-phosphate intermediate. Subsequently, imines or nitroalkenes undergo stereoselective nucleophilic addition with this intermediate to afford the final products. This efficient methodology yields a series of α-amino derivatives **37** with excellent yield (up to 95%) and high stereoselectivity (up to 86:14 *dr*).

**Figure 18:**
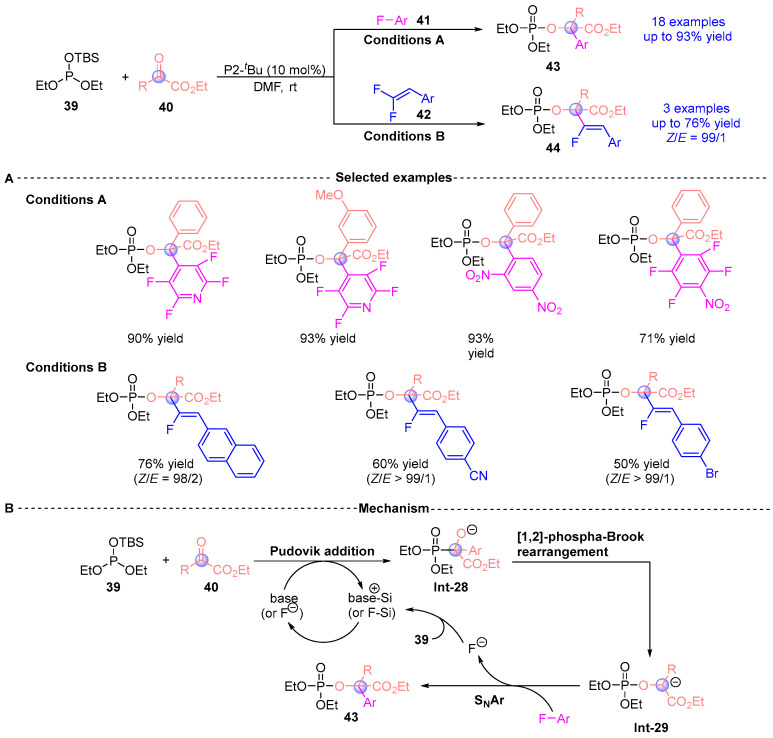
P2-*^t^*Bu-catalyzed [1,2]-phospha-Brook rearrangement reported by Terada. (**A**) Selected examples for **43** and **44**. (**B**) Plausible mechanism.

Inspired by the previous work, Terada [25] reported the synthesis of a series of α-phosphonyloxy ester compounds (**43**, **44**) via a three-component coupling reaction involving silyl phosphites **39**, α-ketoesters **40**, and electron-deficient fluorinated aromatic hydrocarbons (**41**, **42**), catalyzed by P2-*^t^*Bu (Figure 18A). This methodology exhibits excellent substrate scope and regioselectivity, accommodating a wide range of aliphatic and aromatic α-ketoesters without the need for expensive transition metal catalysts. The method enables the introduction of highly electron-deficient aromatic groups, such as polyfluoroaromatic moieties, offering new insights into the development of aromatic nucleophilic reactions. Additionally, the authors proposed a plausible mechanism for the reaction (Figure 18B). Under base or fluoride ion activation, the silyl phosphite **39**, α-ketoester **40**, and fluorinated aromatic hydrocarbon **41** undergo sequential Pudovik addition and [1,2]-phospha-Brook rearrangement to form intermediate **Int-29**, which subsequently proceeds through an S_N_Ar reaction to yield the final product **43**.

**Figure 19:**
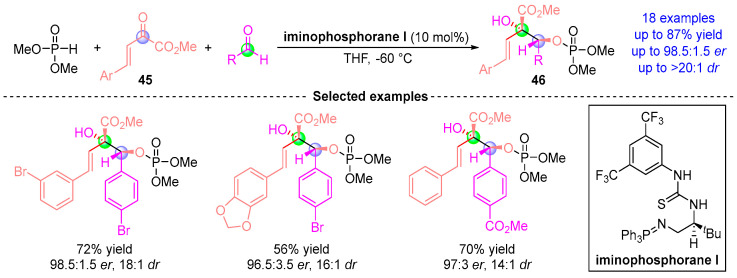
**Iminophosphorane I**-catalyzed [1,2]-phospha-Brook rearrangement reported by Johnson.

In 2016, Johnson et al. [26] reported a three-component asymmetric coupling reaction involving dimethyl phosphite, benzylidenepyruvate **45**, and aldehydes, catalyzed by chiral iminophosphorane (Figure 19). This catalytic system orchestrates a cascade of Pudovik addition and [1,2]-phospha-Brook rearrangement between dimethyl phosphite and benzylidenepyruvate **45** to form enol ester intermediates. Subsequently, these intermediates undergo stereoselective nucleophilic addition with aldehydes, delivering chiral central compound **46** with excellent chemical yield (up to 87%) and high stereoselectivity (up to 97% ee, *dr* > 20:1).

**Figure 20:**
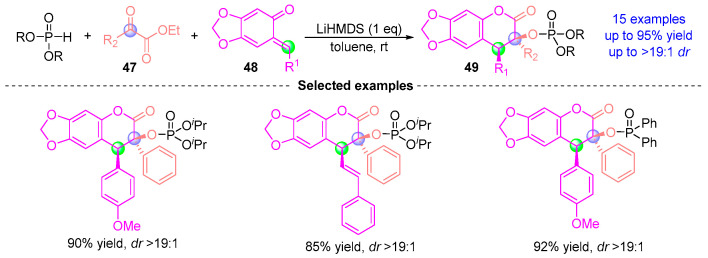
LiHMDS-mediated [1,2]-phospha-Brook rearrangement reported by Sigh.

In 2021, Sigh [27] developed a one-pot catalytic asymmetric synthesis of phosphonate-derived coumarins via a LiHMDS-mediated [1,2]-phospha-Brook/Michael/cyclization cascade (Figure 20). This strategy involves: (i) stereoselective [1,2]-phospha-Brook rearrangement of α-ketoester **47** to generate an enol-phosphate intermediate, (ii) *aza*-Michael addition with *o*-quinone methide **48**, and (iii) rate-determining intramolecular lactonization, affording coumarins **49** with exceptional diastereoselectivity (*dr* > 19:1) and high yields (up to 95%). Notably, this work represents the first catalytic asymmetric synthesis of phosphate-derived coumarins through the direct coupling of phosphoryloxyacetic acid derivatives with *ortho*-quinone methides.

**Figure 21:**
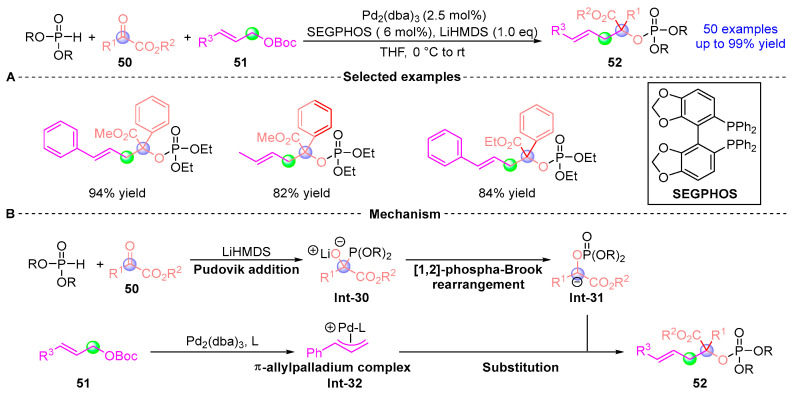
Pd-catalyzed [1,2]-phospha-Brook rearrangement reported by Deng. (**A**) Selected examples for **52**. (**B**) Plausible mechanism.

In 2023, Deng [28] developed a Pd-catalyzed allylic alkylation reaction that synthesizes a series of phosphoketoesters **52** via a three-component tandem reaction involving α-ketoester **50**, β-ketoacid **51**, and dialkyl phosphite (Figure 21A). This protocol is operationally simple and proceeds under mild conditions, exhibiting excellent chemical and regioselectivity with a broad substrate scope. A total of 50 phosphoketoesters with diverse potential biological activities were successfully synthesized with yields up to 99%. Additionally, the authors conducted preliminary studies on the asymmetric synthesis of this reaction and found that using chiral bisphosphine ligands resulted in the target product with a yield of 71% and an ee value of 30%, laying the foundation for future asymmetric catalysis. The proposed mechanism (Figure 21B) involves the following sequence: Diethyl phosphite is first protonated by LiHMDS to form an intermediate, which undergoes Pudovik addition with α-keto acid **50** to generate **Int-30**. Subsequently, **Int-30** undergoes a [1,2]-Phospha-Brook rearrangement to reverse the carbonyl polarity and generate the nucleophilic intermediate **Int-31**. The in situ generated Pd(0)-ligand complex then participates in oxidative addition with **Int-31** to form the π-allylpalladium intermediate **Int-32**. Finally, **Int-32** delivers a nucleophilic attack on **Int-31**, yielding the target product **52** while regenerating the palladium catalyst to perpetuate the catalytic cycle.

**Figure 22:**
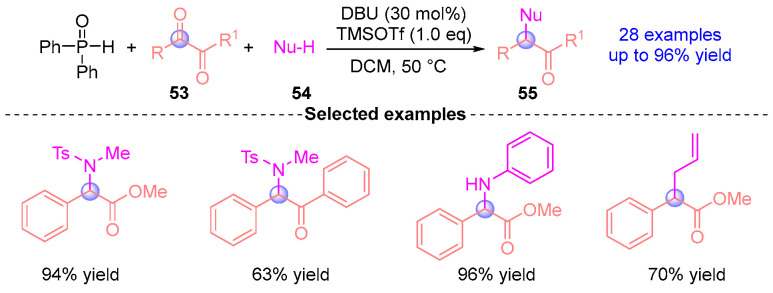
DBU-catalyzed [1,2]-phospha-Brook rearrangement reported by Yamaguchi.

Very recently, the Yamaguchi group [29] developed a deoxygenative functionalization strategy for aromatic dicarbonyl compounds **53** through sequential treatment with DBU, TMSOTf (trimethylsilyl triflate), diphenylphosphine oxide, and nucleophilic reagents **54** to afford functionalized products **55** (Figure 22). This methodology establishes a sequential [1,2]-phospha-Brook rearrangement/benzylic substitution protocol for converting aromatic aldehydes into deoxygenative products. Notably, the reaction proceeds efficiently in the absence of TMSOTf when employing highly nucleophilic reagents. Furthermore, Yamaguchi has extended this approach to enable transformations of diaryl ketones into heterofunctionalized and carbon-functionalized diaryl methanes [30].

## 5. Ketoamide Rearrangement

Ketoamide derivatives demonstrate versatile reactivity profiles owing to their unique combination of nucleophilic and electrophilic sites, complemented by the structural diversity of amide substituents. This distinctive structural characteristic establishes them as privileged substrates for diverse applications, including asymmetric organocatalysis, multifunctional molecular construction, pharmacophore development, and natural product derivatization. Investigating their reactivity patterns in [1,2]-phospha-Brook rearrangements, particularly with respect to stereoelectronic effects and chemodivergent pathways, carries significant implications for the advancement of phosphorus-containing molecular architectures.

### 5.1. Ketoamide Rearrangement with Phosphodiesters

**Figure 23:**
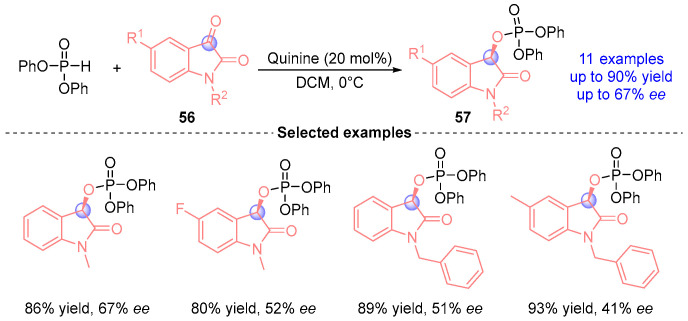
Quinine-catalyzed [1,2]-phospha-Brook rearrangement reported by Wang.

In 2011, Wang’s group [31] achieved the first catalytic asymmetric reaction between diphenyl phosphite and indigo derivatives using a quinine-based catalyst, affording α-phosphoryloxy ester compounds **57** containing an indigo core (Figure 23). This pioneering methodology demonstrated excellent yield and good stereoselectivity in constructing chiral phosphorus-containing heterocycles.

**Figure 24:**
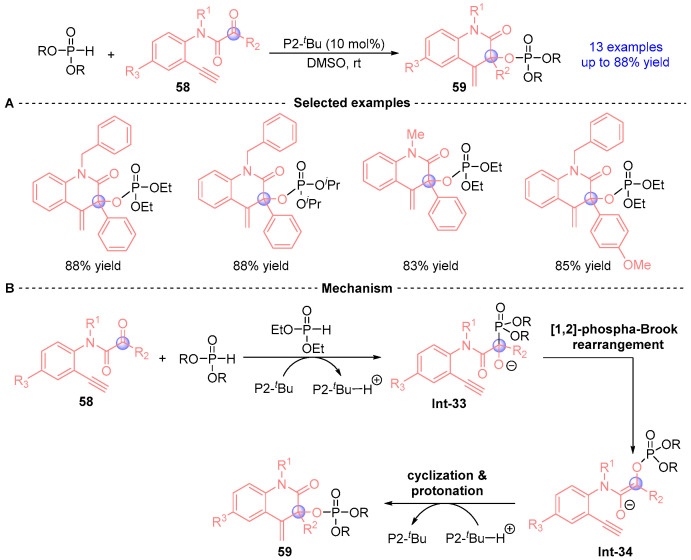
P2-*^t^*Bu-catalyzed [1,2]-phospha-Brook rearrangement reported by Terada. (**A**) Selected examples for **59**. (**B**) Plausible mechanism.

In 2014, Terada’s group [32] developed a catalytic cyclization strategy employing alkynyl α-ketoanilines through [1,2]-phospha-Brook rearrangement, demonstrating broad substrate scope and high efficiency (Figure 24A). The methodology utilizes P2-*^t^*Bu as a catalytic base, where ketoamide **58** undergoes a cascade process through Pudovik addition followed by [1,2]-phospha-Brook rearrangement to generate intermediate **Int-34**. This intermediate subsequently undergoes intramolecular alkyne addition to afford product **59**, as outlined in Figure 24B.

**Figure 25:**
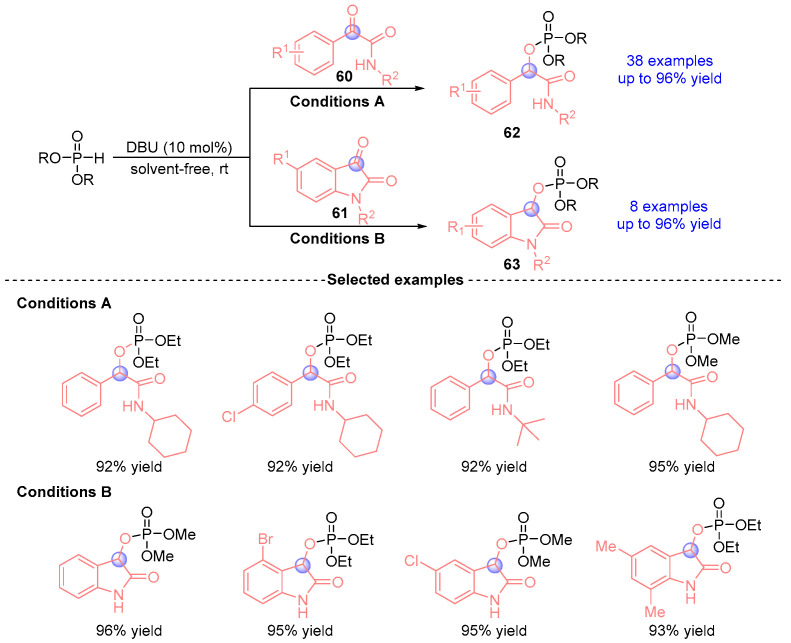
DBU-catalyzed [1,2]-phospha-Brook rearrangement reported by Wu.

In 2020, Wu [33] reported a DBU-catalyzed [1,2]-phospha-Brook rearrangement between α-ketoamides (**60**, **61**) and phosphites under solvent-free conditions (Figure 25). This rapid protocol operates efficiently at room temperature with remarkably short reaction times (2–5 min), delivering 46 structurally diverse analogs (**62**, **63**) in excellent yields (up to 96%). The methodology exhibits broad substrate compatibility with various phosphite derivatives and α-ketoamide substrates. Notably, the process demonstrates scalability to molar quantities while maintaining high efficiency (>91% yield within 5 min).

### 5.2. Ketoamide Rearrangement with Three Components

**Figure 26:**
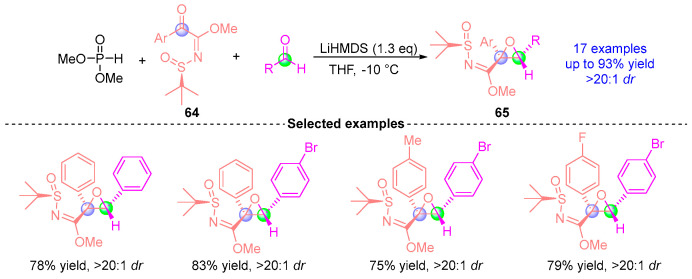
LiHMDS-mediated [1,2]-phospha-Brook rearrangement reported by Xu.

In 2016, Xu’s group [34] developed an efficient asymmetric synthesis strategy for chiral epoxy ternary compounds through a [1,2]-phospha-Brook rearrangement-mediated three-component reaction (Figure 26). This catalytic system employed *N*-*tert*-butylsulfonyl α-ketoimine ester **64**, dimethyl phosphite, and aromatic aldehydes as substrates under LiHMDS mediation. The reaction demonstrated remarkable stereochemical control, delivering 17 structurally diverse chiral epoxy products **65** in yields up to 93% with excellent diastereoselectivity (>20:1 *dr*). This methodology established a practical approach for constructing highly functionalized epoxy architectures through precise manipulation of the [1,2]-phospha-Brook rearrangement pathway.

**Figure 27:**
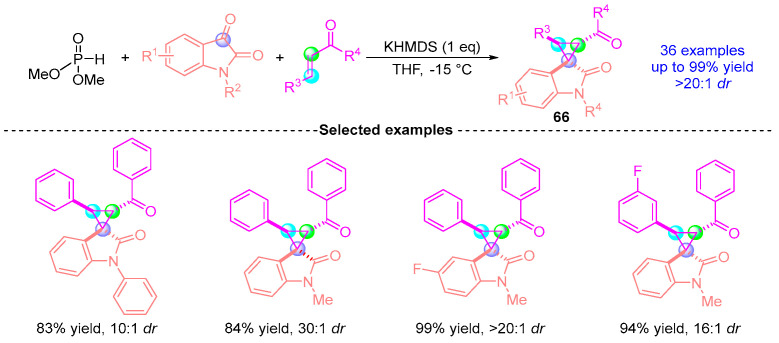
KHMDS-promoted [1,2]-phospha-Brook rearrangement reported by Xu.

Subsequently, Xu’s group [23] in 2017 extended their methodology to achieve an asymmetric [1,2]-phospha-Brook rearrangement involving indigo carmine-derived substrates, α,β-unsaturated ketones, and dimethyl phosphite (Figure 27). Employing KHMDS (potassium hexamethyldisilazide) as the catalytic base, this three-component reaction enabled the stereocontrolled synthesis of highly functionalized cyclopropane derivative **66**, which was obtained in 99% yield with excellent diastereoselectivity (>20:1 *dr*). The protocol demonstrated broad substrate compatibility while retaining mechanistic parallels to the group’s 2016 system, with diastereoselectivity primarily governed by substrate electronic effects and precise temperature modulation.

**Figure 28:**
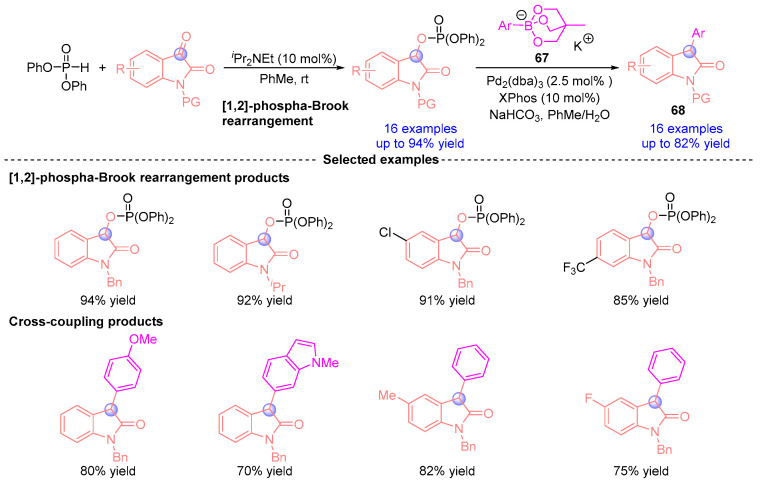
*^i^*Pr_2_NEt-catalyzed [1,2]-phospha-Brook rearrangement reported by Terada.

In 2016, Terada and coworkers [35] developed a streamlined protocol for synthesizing 3-arylindole derivatives from indigo carmine-derived substrates (Figure 28). The process begins with an *^i^*Pr_2_NEt-catalyzed [1,2]-phospha-Brook rearrangement, generating a C3-phosphorylated indole oxide intermediate. Subsequent palladium-catalyzed cross-coupling with arylboron reagent **67** enabled one-pot construction of 3-aryloxyindole derivatives **68**. Mechanistically, the transformation proceeds through α-phosphoryloxy ester intermediates, followed by sequential phosphonyl group elimination and aryl coupling to assemble unique molecular architectures, establishing a novel platform for complex heterocycle synthesis.

**Figure 29:**
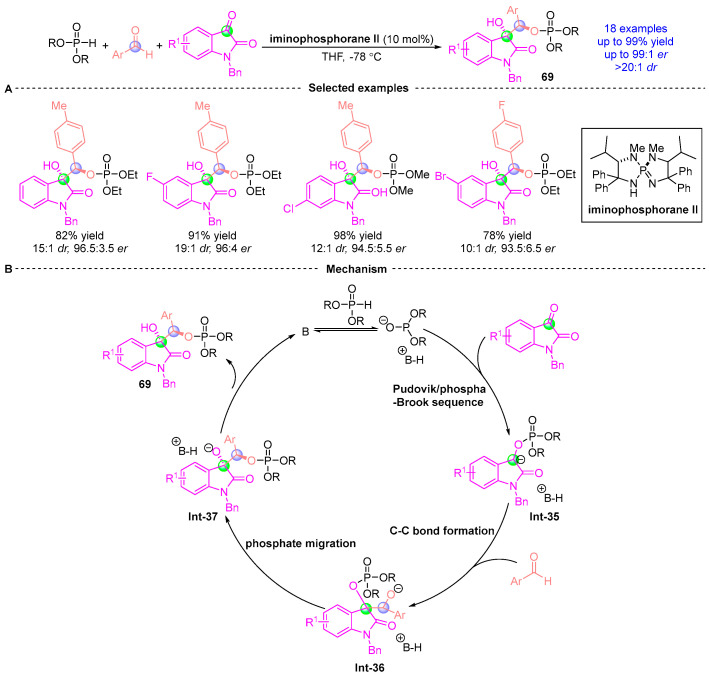
Iminophosphorane II-catalyzed [1,2]-phospha-Brook rearrangement reported by Oil. (**A**) Selected examples for **69**. (**B**) Plausible mechanism.

In 2015, Ooi [36] demonstrated a stereoselective three-component assembly of phosphodiesters, indigo-derived substrates, and aromatic aldehydes (Figure 29A). This catalytic asymmetric process delivered structurally diverse indigo-containing chiral compounds **69** with exceptional efficiency (up to 99% yield, 99:1 *er*, and >20:1 *dr*). Mechanistically, the transformation initiates with a Pudovik addition/[1,2]-phospha-Brook rearrangement cascade between indigo derivatives and phosphodiesters, generating chiral α-phosphoryloxy intermediate **Int-35**. Subsequent deprotonation forms a carbanion species that undergoes phosphoimine-catalyzed nucleophilic addition with aromatic aldehydes to yield intermediate **Int-36**. Phosphate group migration then produces **Int-37**, ultimately furnishing the target indigo-embedded chiral architectures through sequential bond reorganization (Figure 29B). This work established a powerful strategy for constructing complex indigo-based chiral systems via orchestrated phosphorus-mediated transformations.

**Figure 30:**
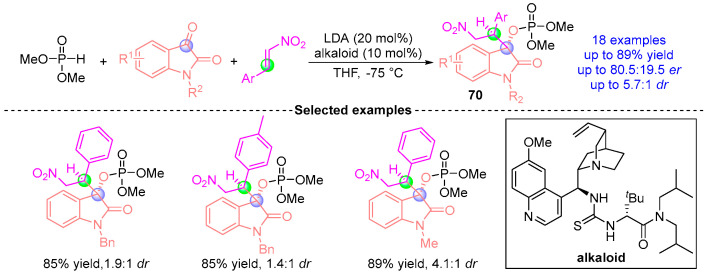
LDA/alkaloid-catalyzed [1,2]-phospha-Brook rearrangement reported by Johnson.

Based on previous work experience, Johnson et al. [37] 2017 advanced the catalytic asymmetric coupling of indigo carmine-derived substrates with nitroalkenes and phosphodiesters (Figure 30). Employing a chiral quinine-derived thiourea catalyst with LDA (lithium diisopropylamide) in THF at −75 °C, this system generated 18 β-nitroindigo derivatives **70** through stereodivergent synthesis. The protocol achieved 89% yield with 83.5:16.5 *dr*, demonstrating precise stereochemical control predominantly governed by the thiourea catalyst’s chiral microenvironment. This work significantly expanded the synthetic utility of indigo frameworks in asymmetric transformations while maintaining operational simplicity under cryogenic conditions.

**Figure 31:**
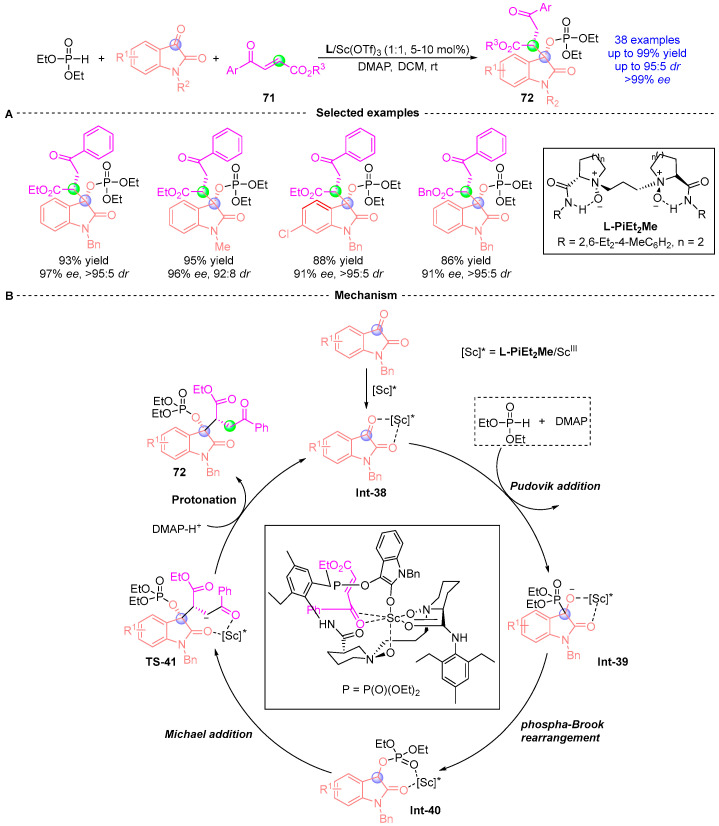
L-PiEt_2_Me/Sc-catalyzed [1,2]-phospha-Brook rearrangement reported by Feng. (**A**) Selected examples for **72**. (**B**) Plausible mechanism.

In 2023, Feng [38] achieved a stereodivergent synthesis of 3,3-disubstituted indolone derivatives **72** bearing two chiral centers through a chiral diazo/scandium-catalyzed cascade process combining [1,2]-phospha-Brook rearrangement and phospha-Michael addition (Figure 31A). This operationally simple protocol demonstrated remarkable stereocontrol (up to 99% yield, 99% *ee*, and 95:5 *dr*) under mild conditions with broad substrate generality, accommodating diverse indigo carmine derivatives, and γ-carbonyl butenoates bearing aryl, alkyl, or ester substituents. Mechanistic studies revealed the critical importance of sequential substrate addition and chiral Lewis acid mediation. The initial combination of hypophosphite and indigo substrates generates the α-phosphoryloxy intermediate through Pudovik addition/[1,2]-phospha-Brook rearrangement, which subsequently undergoes catalyst-controlled phospha-Michael addition with enone esters. Concurrent mixing of all three substrates proved ineffective for product formation. The proposed mechanism (Figure 31B) involves chiral *L*-PiEt_2_Me/Sc complex activation of the indigo substrate, followed by DMAP (4-dimethylaminopyridine) mediated generation of enol ester intermediate **Int-40** via sequential Pudovik addition and phospha-Brook rearrangement with diethyl phosphite. Stereoselective *Re*-face attack of **Int-40**, directed by the diazo/scandium catalyst, forms Michael adduct **Int-41**. Final protonation yields the doubly stereogenic product **72** with defined (*S,R*)-configuration.

**Figure 32:**
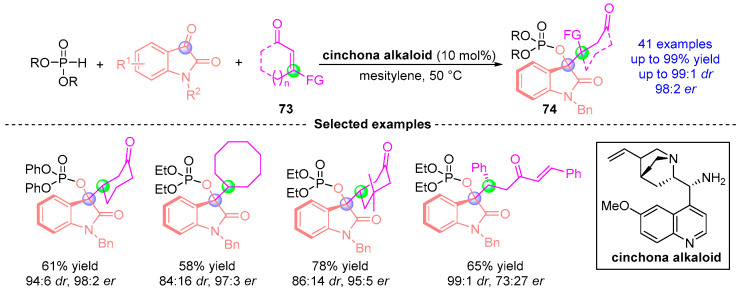
Cinchona alkaloid-catalyzed [1,2]-phospha-Brook rearrangement reported by Sigh.

Very recently, Sigh and coworkers [39] disclosed a catalytic asymmetric cascade sequence combining Pudovik addition, [1,2]-phospha-Brook cyclization, and Michael reaction using a bifunctional aminocatalytic system (Figure 32). This three-component strategy effectively converts indigo carmine derivatives, dialkyl phosphites, and α,β-unsaturated ketones **73** into indole derivatives **74** bearing vicinal stereocenters. The catalytic system operates through a dual activation mechanism: imine-mediated enol activation synergizes with Brønsted base-promoted carbanion stabilization. Remarkably, this additive-free protocol demonstrates broad functional group tolerance under mild conditions while achieving excellent chemoselectivity and stereocontrol (up to 98:2 *er*, 99:1 *dr*). The method’s synthetic potential is further highlighted by its scalability and versatile downstream transformations of the phospho-indole products.

## 6. Ketene Rearrangement

The [1,2]-phospha-Brook rearrangement enables the efficient transformation of enones into enol-phosphate-versatile synthons that play pivotal roles in drug discovery, functional material fabrication, and biomolecular engineering.

**Figure 33:**
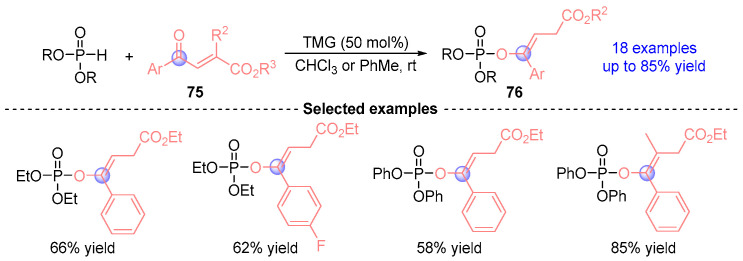
TMG-catalyzed [1,2]-phospha-Brook rearrangement reported by Xiao.

In 2012, Xiao [40] developed a TMG (tetramethylguanidine)-catalyzed [1,2]-phospha-Brook rearrangement protocol for synthesizing enol phosphates **76** from phosphodiesters and carbonyl esters **75** (Figure 33). This practical method delivered 17 structurally diverse enol-phosphate derivatives with up to 85% yield, highlighting operational simplicity and compatibility with commercially available hypophosphite reagents.

**Figure 34:**
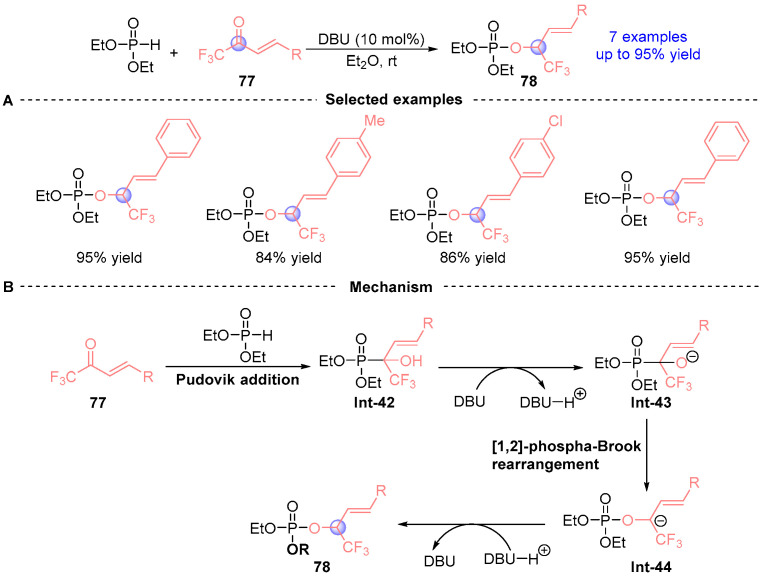
DBU-catalyzed [1,2]-phospha-Brook rearrangement reported by Liu. (**A**) Selected examples for **78**. (**B**) Plausible mechanism.

In 2017, Liu [41] established a catalyst-dependent divergent synthesis using α,β-unsaturated trifluoromethyl ketones **77**, and diethyl phosphite (Figure 34A). DABCO (1,4-diazabicyclo[2.2.2]octane) catalysis induced selective Pudovik addition under mild conditions to furnish α-hydroxyphosphonate **Int-42**, while DBU promoted sequential Pudovik addition/[1,2]-phospha-Brook rearrangement, ultimately delivering α-phosphoryloxy ester **76** via protonation of intermediate **Int-43**. The electron-deficient trifluoromethyl group critically stabilizes transition state **Int-44** during the rearrangement phase (Figure 34B), demonstrating strategic exploitation of electronic effects to control reaction trajectories.

**Figure 35:**
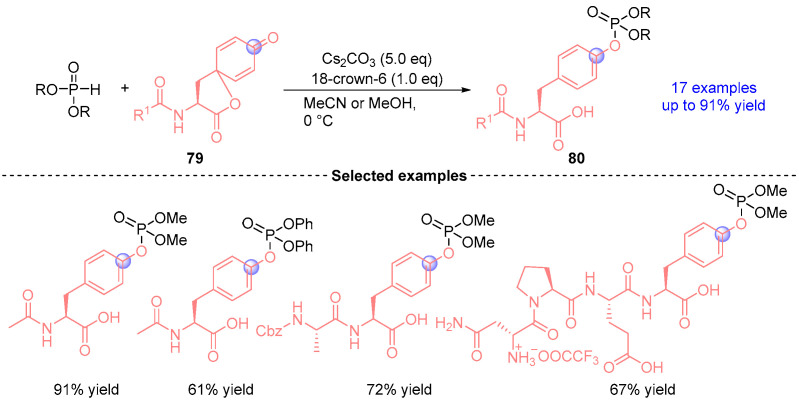
Cs_2_CO_3_-mediated [1,2]-phospha-Brook rearrangement reported by Kanai.

In 2024, Kanai [42] established a Cs_2_CO_3_-promoted 1,2-phospha-Brook rearrangement based on tyrosine-derived spiro-lactones **79** and phosphite diesters to give a wide range of peptide substrates containing diverse nucleophilic amino acid residues, including serine and threonine (Figure 35). Particularly noteworthy is its successful implementation across various secondary structures and complex peptide sequences, addressing longstanding challenges in selective post-translational modification chemistry.

## 7. Alkyne Ketone Rearrangement

The [1,2]-phospha-Brook rearrangement of ynones has emerged as a powerful strategy for generating allenyl intermediates that facilitate stereocontrolled intramolecular cyclization to access five-membered heterocyclic architectures. Notably, Feng and Wang recently extended this paradigm to asymmetric synthesis, achieving the first catalytic construction of axially chiral allenes through precise control of rearrangement dynamics, thereby opening new avenues in chiral molecule synthesis.

**Figure 36:**
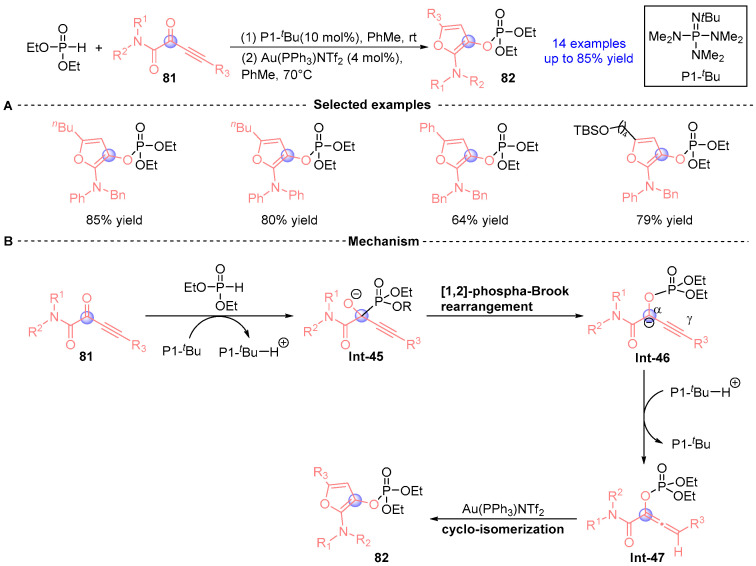
P1-*^t^*Bu/Au-catalyzed [1,2]-phospha-Brook rearrangement reported by Terada. (**A**) Selected examples for **82**. (**B**) Plausible mechanism.

In 2016, Terada's team [43] developed a catalytic cascade strategy for synthesizing 2-amino-3-phosphoryloxyfuran derivatives through sequential [1,2]-phospha-Brook rearrangement and gold-mediated cyclization (Figure 36A). The process initiates with α-alkynyl ketamide **81** undergoing P1-*^t^*Bu-catalyzed Pudovik addition/[1,2]-phospho-Brook rearrangement with phosphodiesters to form bicyclic intermediate **Int-46**. Subsequent protonation generates **Int-47**, which undergoes gold-catalyzed stereoselective intramolecular cyclization to furnish phosphorylated furan **82** (Figure 36B). Notably, thermal activation of **82** in the absence of gold induces intramolecular Diels-Alder cyclization, selectively producing six-membered diene architectures.

**Figure 37:**
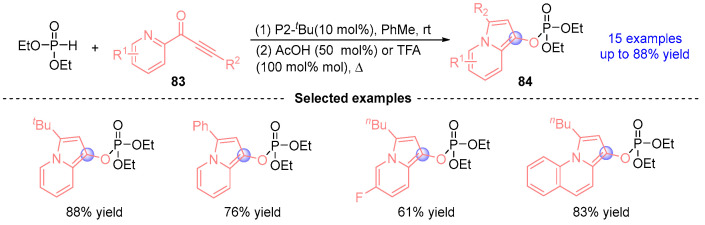
P2-*^t^*Bu/acid-catalyzed [1,2]-phospha-Brook rearrangement reported by Terada.

Based on previous work, Terada [44] advanced their [1,2]-phospha-Brook methodology by developing a transition metal-free protocol for indole synthesis through P2-*^t^*Bu-catalyzed [1,2]-phospha-Brook rearrangement (Figure 37). This operationally straightforward method efficiently constructed indole-pyrimidine derivatives **84** with good yields, demonstrating broad functional group compatibility. Furthermore, the system exhibited remarkable versatility nickel mediated arylative dephosphorylation of the phosphorylated intermediates with Grignard reagents enabled selective C-arylation, establishing a dual functionalization strategy for modular indole architecture construction. This cascade approach significantly expands the synthetic toolbox for heterocyclic compound diversification without requiring precious metal catalysts.

**Figure 38:**
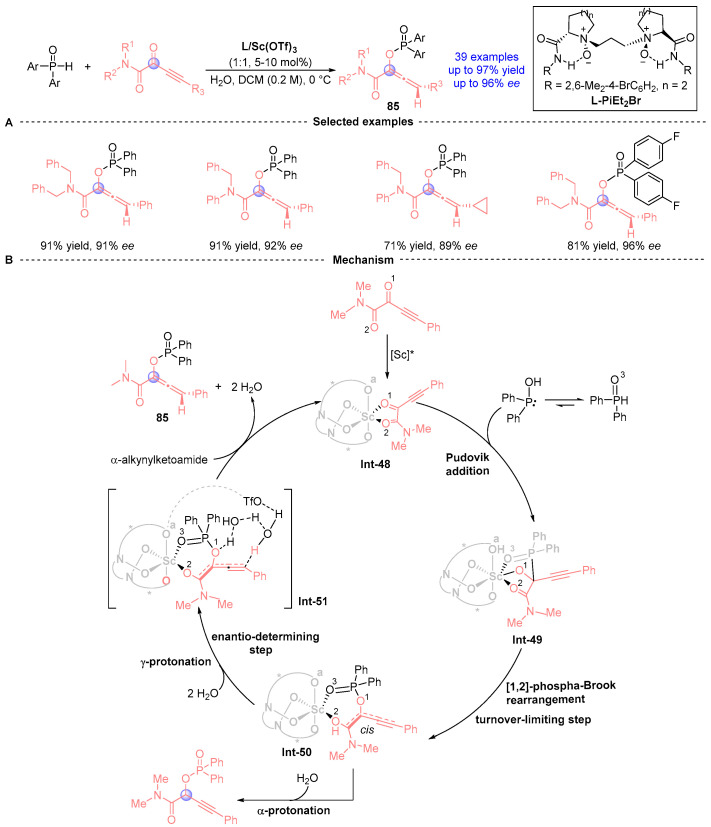
L-PiEt_2_Me/Sc-catalyzed [1,2]-phospha-Brook rearrangement reported by Feng. (**A**) Selected examples for **85**. (**B**) Plausible mechanism.

In 2022, Feng [45] achieved a breakthrough in asymmetric catalysis using their proprietary *N,N′*-dioxide/Sc(III) complex to mediate enantioselective Pudovik addition/[1,2]-phospha-Brook rearrangement cascades between α-alkyl-acyl ketone amides and diarylphosphine oxides (Figure 38A). This catalytic system delivered 36 axially chiral alkenes **85** with exceptional efficiency (up to 97% yield) and stereocontrol (up to 96% *ee*) while demonstrating broad substrate scope and functional group tolerance. The synthetic utility was further highlighted by converting products into complex bridged polycyclic architectures through dearomative cyclization pathways. Mechanistic studies combining experimental and computational methods (Figure 38B) revealed a three-stage process: (i) Scandium-activated ketone amide undergoes Pudovik addition to form tetrahedral intermediate **Int-49**; (ii) Configuration-inverting [1,2]-phospha-Brook rearrangement generates phospho-enolate **Int-50**; and (iii) Water-mediated **Int-51** protonation completes the catalytic cycle. The work elucidated critical ligand field effects and counterion/water participation in controlling regio- and stereoselectivity, establishing fundamental principles for designing asymmetric [1,2]-phospha-Brook rearrangements.

**Figure 39:**
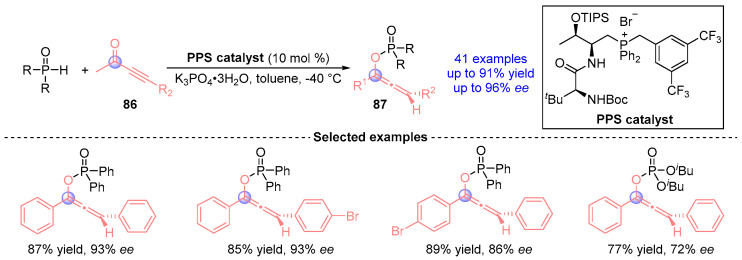
PPS-catalyzed [1,2]-phospha-Brook rearrangement reported by Wang.

Wang [46] established a biomimetic catalytic cascade employing peptide-mimic phosphonium salt as the catalyst for the asymmetric synthesis of axially chiral alkenylphosphines (Figure 39). This transition metal-free platform synergistically combines Pudovik addition and [1,2]-phospha-Brook rearrangement of alkynyl ketones **86** with phosphine oxides, delivering 41 stereodefined phosphoalkene derivatives **87** with exceptional efficiency and enantiocontrol. The methodology’s bioinspired design enables precise stereochemical regulation while maintaining broad functional group compatibility, representing a significant advance in organocatalytic phosphorus chemistry.

## 8. Multicomponent Rearrangement

While mechanistic consensus exists regarding the α-hydroxyphosphonate intermediate pathway in [1,2]-phospha-Brook rearrangement, experimental verification using prepared α-hydroxyphosphonates remains limited. This knowledge gap arises from the reaction’s inherent efficiency and the process readily initiates through in situ generation of reactive intermediates from phosphodiesters and carbon nucleophiles, circumventing the requirement for discrete intermediate isolation. This inherent reactivity profile not only demonstrates the transformation’s operational simplicity but also exemplifies its atom-economical nature, underscoring its value in contemporary synthetic methodologies.

**Figure 40:**
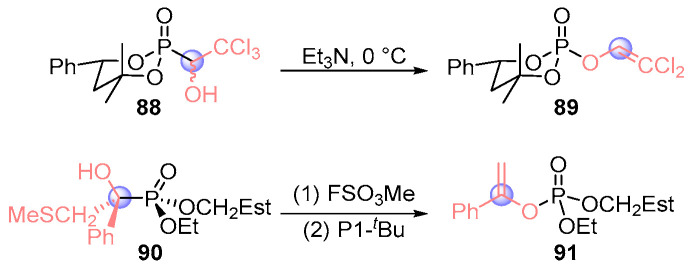
Et_3_N or P1-*^t^*Bu-mediated [1,2]-phospha-Brook rearrangement reported by Hammerschmidt.

Hammerschmidt’s team pioneered stereochemical studies of [1,2]-phospha-Brook rearrangements through sequential investigations. Their 2015 work [47] demonstrated configuration retention in trichlorophosphoryl scaffold **88** under triethylamine mediation, cleanly generating dichloroallyl phosphate **89** while preserving stereochemical integrity (Figure 40). This established a viable route to optically pure enol phosphates through stereospecific transformations. Building on this foundation, then they [48] developed a chiral resolution strategy to obtain enantiopure α-hydroxyphosphate **90**. Sequential methylation with methyl thiofluorosulfate and P1-*t*Bu-mediated rearrangement achieved complete stereochemical transfer, delivering enol phosphate **91** as a single stereoisomer. These systematic investigations fundamentally advanced the understanding of stereochemical fidelity in phospho-Brook processes.

**Figure 41:**
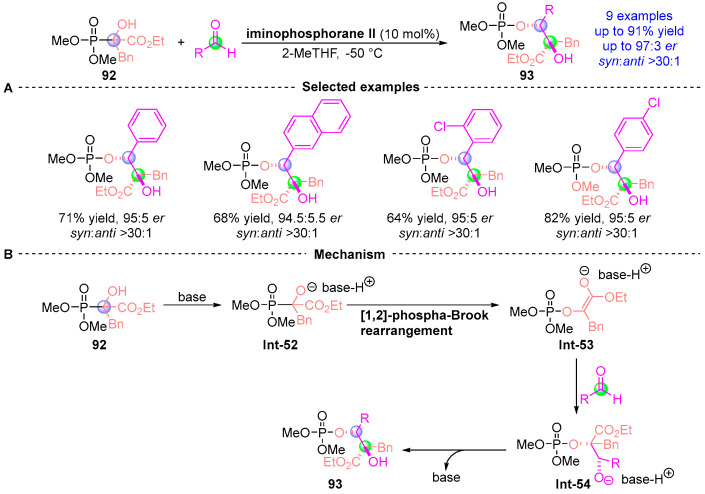
Iminophosphorane II-catalyzed [1,2]-phospha-Brook rearrangement reported by Johnson. (**A**) Selected examples for **93**. (**B**) Plausible mechanism.

In 2012, Johnson et al. [49] developed an asymmetric [1,2]-phospha-Brook rearrangement strategy using α-hydroxy phosphate **92** and aldehydes to construct bis-chiral α-phosphoryloxy derivatives **93** with precise stereochemical control (Figure 41A). This catalytic system demonstrated broad substrate scope across alkyl, vinyl, aryl, and heteroaromatic aldehydes, delivering nine derivatives with excellent diastereocontrol (up to 97:3 *dr*) and yields (up to 91%). The phosphoryl group’s strategic lability enabled subsequent *p*-toluenesulfonic acid-mediated deprotection, establishing a versatile platform for chiral scaffold diversification. Mechanistic analysis (Figure 41B) revealed a concerted process: (i) Catalyst-mediated rearrangement of **92** generates oxaphosphetane intermediate **Int-52**; (ii) Stereoselective aldehyde addition forms **Int-53**; (iii) Phosphoryl migration through **Int-54** precedes stereospecific protonation to finalize **93**. This sequence highlights the methodology’s dual capacity for stereochemical induction and functional group manipulation.

**Figure 42:**
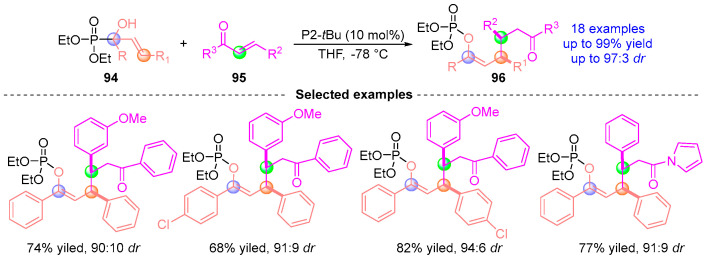
P2-*^t^*Bu-catalyzed [1,2]-phospha-Brook rearrangement reported by Terada.

In 2017, Terada [50] pioneered a catalytic homologation strategy leveraging [1,2]-phospha-Brook rearrangement to generate allylic anions from phosphate ester **94** and α-hydroxyallyl-functionalized chalcone **95** (Figure 42). This transition metal-free protocol enabled stereoselective C–C bond formation, delivering chiral compounds **96** with excellent yields and stereocontrol. The methodology capitalizes on the inherent nucleophilicity of [1,2]-phospho-Brook intermediates while maintaining precise stereochemical fidelity, exemplifying a novel approach to constructing complex allylic architectures through anion relay chemistry.

**Figure 43:**
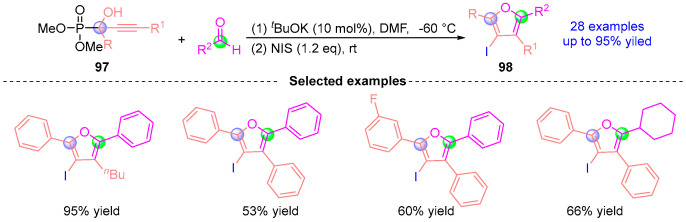
*^t^*BuOK-catalyzed [1,2]-phospha-Brook rearrangement reported by Terada.

In 2020, Terada’s group [51] developed a *^t^*BuOK-catalyzed cascade synthesis of tetrasubstituted furans through [1,2]-phospha-Brook rearrangement/cyclization sequences (Figure 43). The one-pot protocol initiates with α-hydroxy phosphate **97** undergoing base-mediated rearrangement to generate a reactive carbanion intermediate. This species subsequently engages aldehydes in NIS (*N*-iodosuccinimide) promoted [3 + 2] annulation, delivering 2,4,5-trisubstituted 3-iodofurans **98** that serve as versatile precursors to fully substituted furan architectures. Demonstrating exceptional functional group tolerance and step economy, this methodology establishes a robust platform for constructing complex heterocyclic systems through synergistic anion generation and electrophilic trapping strategies.

**Figure 44:**
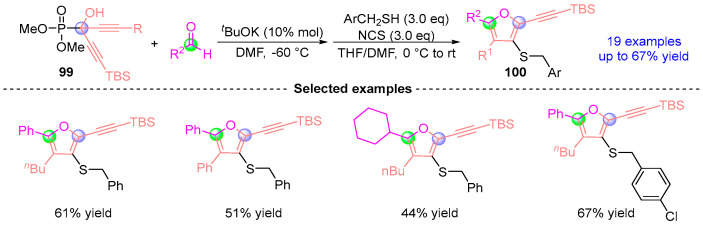
*^t^*BuOK-catalyzed [1,2]-phospha-Brook rearrangement reported by Terada.

Based on previous work, Terada [52] achieved a breakthrough in heterocycle synthesis through a programmable two-phase assembly of thieno[3,2-b]furan architectures (Figure 44). This platform synergistically merges [1,2]-phospha-Brook rearrangement-driven [3 + 2] cyclofunctionalization with Brønsted base enabled annulative closure, enabling efficient construction of highly substituted 2,3,5,6-tetrasubstituted thieno[3,2-b]furans targets **100** traditionally inaccessible through conventional methods. The methodology demonstrates exceptional generality across diverse substrates while maintaining precise regiocontrol, with successful adaptation to seleno[3,2-b]furan systems further validating its versatility as a heterocyclic construction platform.

**Figure 45:**
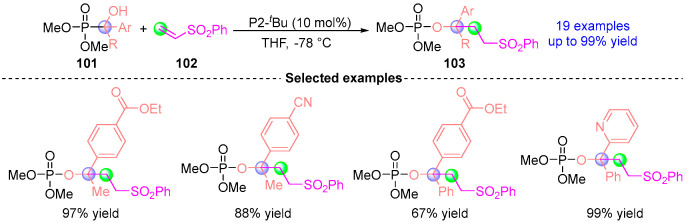
P2-*^t^*Bu-catalyzed [1,2]-phospha-Brook rearrangement reported by Terada.

Concurrently, they [53] devised a polarity-reversal strategy for tertiary alcohol synthesis through Brønsted base-mediated umpolung functionalization (Figure 45). This innovative approach leverages α-hydroxyphosphonates **101** (derived from aryl ketones) to engage electrophiles like phenyl vinyl sulfone **102** via [1,2]-phospha-Brook rearrangement, forging tertiary alkyl-containing phosphates **103** with precise stereochemical control. Subsequent hydrolysis of these bench-stable intermediates provides streamlined access to sterically congested benzylic tertiary alcohol structures historically challenging to construct through classical nucleophilic pathways. The methodology’s synthetic power stems from its ability to invert conventional reactivity paradigms while maintaining operational simplicity, establishing a novel disconnection for three-dimensional molecular architectures.

**Figure 46:**
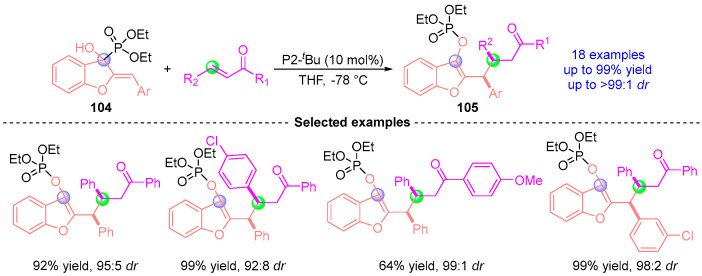
P2-*^t^*Bu-catalyzed [1,2]-phospha-Brook rearrangement reported by Terada.

Recently, Terada [54] also demonstrated the synthetic versatility of Brønsted base-catalyzed [1,2]-phospha-Brook rearrangement through an innovative intermolecular coupling platform (Figure 46). Employing phosphazene superbase P2-*^t^*Bu, the methodology generates bench-stable benzofuran-anchored diarylmethyl anions **104** via rearrangement-induced deprotonation. These configurationally defined carbanions exhibit broad electrophile compatibility, engaging α,β-unsaturated ketones, and related partners to deliver structurally complex diarylalkanes **105** with excellent yield (up to 99%) and precise stereochemical governance (up to 99:1 *dr*).

## 9. Conclusions

In summary, the [1,2]-phospha-Brook rearrangement has emerged as a powerful paradigm for generating configurationally defined anionic nucleophiles through carbonyl group activation. Over two decades of development, this transformation has evolved into a versatile platform compatible with diverse carbonyl substrates including aldehydes, ketones, ketoesters, ketimines, alkynones, and α-hydroxyphosphates. What is more, the inherent capacity to generate stabilized carbanion intermediates enables efficient assembly of versatile motifs through subsequent regioselective functionalization even in both diastereo- and enantioselective pathways. In the future, the [1,2]-phospha-Brook rearrangement might focus on the development of novel catalytic systems, stereodivergent synthetic strategies, and mechanism-driven innovations in catalytic modes.

## Data Availability

Not Applicable.

## Abbreviations

The following abbreviations were used in this manuscript:

AChE	Acetylcholinesterase
BChE	Butyrylcholinesterase
BPR	Berry pseudorotation
CaE	Carboxylesterases
DABCO	1,4-Diazabicyclo[2.2.2]octane
DBU	1,8-Diazabicyclo [5.4.0]undec-7-ene
DMAP	4-Dimethylaminopyridine
DMF	*N,N*-Dimethylformamide
DFT	Density functional theory
KHMDS	Potassium Hexamethyldisilazide
LDA	Lithium Diisopropylamide
LiHMDS	Lithium Hexamethyldisilazide
NaHMDS	Sodium Hexamethyldisilazide
NIS	*N*-Iodosuccinimide
PhOK	Potassium phenoxide
SPO	Secondary phosphine oxides
TMG	Tetramethylguanidine
TMSOTf	Trimethylsilyl triflate
*^n^*BuLi	*n*-butyllithium
*^t^*BuOK	potassium tert-butoxide
